# Brown Norway Chromosome 1 Congenic Reduces Symptoms of Renal Disease in Fatty Zucker Rats

**DOI:** 10.1371/journal.pone.0087770

**Published:** 2014-01-31

**Authors:** Craig H. Warden, Carolyn Slupsky, Stephen M. Griffey, Ahmed Bettaieb, Esther Min, Anh Le, Janis S. Fisler, Susan Hansen, Fawaz Haj, Judith S. Stern

**Affiliations:** 1 Department of Pediatrics and Department of Neurobiology, Physiology and Behavior, School of Veterinary Medicine, University of California, Davis, California, United States of America; 2 Department of Nutrition, School of Veterinary Medicine, University of California, Davis, California, United States of America; 3 Comparative Pathology Laboratory, School of Veterinary Medicine, University of California, Davis, California, United States of America; 4 Department of Internal Medicine, University of California, Davis, California, United States of America; Emory University, United States of America

## Abstract

We previously reported that a congenic rat with Brown Norway (BN) alleles on chromosome 1 reduces renal disease of 15-week old fatty Zucker rats (ZUC). Development of renal disease in fatty BN congenic and fatty ZUC rats from 9 through 28 weeks is now examined. Analysis of urine metabolites by ^1^H nuclear magnetic resonance (NMR) spectroscopy revealed a significantly increased urinary loss of glucose, myo-inositol, urea, creatine, and valine in ZUC. Food intake was lower in the BN congenic rats at weeks 9–24, but they weighed significantly more at 28 weeks compared with the ZUC group. Fasting glucose was significantly higher in ZUC than congenic and adiponectin levels were significantly lower in ZUC, but there was no significant genotype effect on Insulin levels. Glucose tolerance tests exhibited no significant differences between ZUC and congenic when values were normalized to basal glucose levels. Quantitative PCR on livers revealed evidence for higher gluconeogenesis in congenics than ZUC at 9 weeks. Plasma urea nitrogen and creatinine were more than 2-fold higher in 28-week ZUC. Twelve urine protein markers of glomerular, proximal and distal tubule disease were assayed at three ages. Several proteins that indicate glomerular and proximal tubular disease increased with age in both congenic and ZUC. Epidermal growth factor (EGF) level, a marker whose levels decrease with distal tubule disease, was significantly higher in congenics. Quantitative histology of 28 week old animals revealed the most significant genotype effect was for tubular dilation and intratubular protein. The congenic donor region is protective of kidney disease, and effects on Type 2 diabetes are likely limited to fasting glucose and adiponectin. The loss of urea together with a small increase of food intake in ZUC support the hypothesis that nitrogen balance is altered in ZUC from an early age.

## Introduction

Zucker fatty rats have been established as a model for renal disease and type 2 diabetes [1]–[3]. Zucker fatty rats (ZUC- *Lepr^faSte^* (RGD ID: 629462) referred to as ZUC or the ZUC strain in this paper) exhibit extreme obesity when they are homozygous for the recessive fatty mutation in the leptin receptor (*Lepr^faSte^*). ZUC homozygous for *Lepr^faSte^* will be referred to as fatty ZUC for the remainder of this paper. ZUC either homozygous or heterozygous for the wildtype allele (*Lepr^+^* or *Lepr^faSte/+^*) are lean and are designated lean ZUC in this paper. Previous studies of ZUC rats also reported that hyperphagia and hypertriglyceridemia accelerate renal disease [4], [5]. However, studies comparing lean and obese ZUC rats cannot distinguish between effects caused by obesity due to the chromosome 4 *Lepr* mutation from those caused by an interaction of *Lepr* with specific alleles from other chromosomes such as obesity-dependent renal and type 2 diabetes alleles.

We previously demonstrated that Brown Norway (BN/Crl (RGD ID: 737972)) chromosome 1 reduces type 2 diabetes and renal disease of ZUC fatty rats [1]. In a subsequent study we reported production of a congenic and mapping of renal disease related traits within the congenic donor region [6]. A congenic strain is identical to a background strain except that the congenic has a chromosome, or portion of a chromosome, from a donor strain with all remaining chromosomal DNA from the background strain. Phenotype differences between congenic and background strains are due to differences between donor and background strain alleles. The congenic strain we produced (ZUC.BN-(*D1Rat42-D1Rat90*)/Ste, referred to as ZUC.BN-Chr1 or as the congenic strain in the rest of this paper), has BN alleles for the middle and distal half of chromosome 1. ZUC.BN-Chr1 congenics homozygous for *Lepr^faSte^* will be referred to as fatty congenics. ZUC.BN-Chr1 congenics homozygous or heterozygous for the wildtype allele (*Lepr^+^* or *Lepr^faSte/+^*) are lean and are designated lean congenic in this paper.

We mapped renal disease phenotypes to specific regions of chromosome 1 using an F2 intercross of the congenic and ZUC [6]. We produced animals with all variations of the BN donor region; homozygous BN for the entire donor region, homozygous ZUC for the entire donor region, and all combinations of heterozygous and recombinant donor region chromosomes. We also studied lean and fatty animals with all the donor region genotypes. These studies allowed us to determine which phenotypes were directly caused by obesity and which required an interaction of obesity with alleles on chromosome 1. Our results suggested that interactions of *Lepr* with chromosome 1 occurred for many traits.

Studies of congenic rats have identified genes that cause renal disease, such as *Arhgef11* [7], and *Rab38* which is located on chromosome 1 within the donor region of the ZUC.BN-Chr1 congenic [8]. Other investigators also demonstrated that another gene in the ZUC.BN-Chr1 donor region, *Sorcs1*, causes renal disease in both rats and humans [9].

We have now used the lean and fatty ZUC.BN-Chr1 congenic and lean and fatty ZUC to determine mechanisms for renal disease. Unlike comparisons of lean to obese ZUC rats, these studies allow us to determine which traits are caused by obesity and which require permissive genotypes from the chromosome 1 BN donor region.

## Methods

### Ethics statement

All protocols followed the guidelines of the American Association for Accreditation of Laboratory Animal Care (AAALAC, approval number 000029) and were approved by the Institutional Animal Care and Use Committee (IACUC) of the University of California, Davis (Davis, CA).

### Animals

Animals were cared for in a conventional facility. Rats were housed in polycarbonate caging (unless food intake and urine were being collected) with ad libitum access to food (PMI LabDiet, #5008 Formulab Diet) and deionized water. Both groups (congenics and ZUC) were housed with a temperature range of 70–72°F, humidity 50–60%, and a light/dark cycle of 14/10. At time of sacrifice, animals were fasted overnight, anesthetized with isoflurane, and blood removed by cardiac puncture. Rats were euthanized by overdose of isoflurane, exsanguination, and by cutting of the diaphragm. Tissues were then taken for analysis.

Congenic animals for phenotyping were constructed as described previously [6]. The congenic colony is homozygous BN from *D1Rat42* to *D1Rat90* and is heterozygous for *Lepr^faSte/+^*. Intercrosses of these animals produce progeny where 25% are wildtype and 25% are homozygous fatty for *Lepr^faSte^*.

### Assays

Fasted EDTA plasma was collected at sacrifice as described [10]. For urine collection, rats were weighed then individually housed in wire metabolic caging with fitted wire mesh over funnels for 24 hours. Rats were given ad libitum access to wet mash using a ground meal version of their normal chow and water. Glass wool was placed in the tip of the funnel to minimize contamination from food particles and feces. Three drops of 10% sodium azide solution was added to each sample beaker to suppress bacterial growth. Urine was collected after 24 hours. Urine volume was measured, then centrifuged and aliquoted. UAE (mg/24 hours) was determined by the albumin blue 580 method [11], [12].

Food intake was measured by placing rats individually onto wire bottom cages. Body weight, food given, food left, and spill were measured.

Assays were as described previously [6], with the following additions. KidneyMAP assays for urine proteins were conducted by Myriad Genetics, whereas assays of plasma metabolites and enzymes were performed by the UC Davis Comparative Pathology Laboratory. Insulin was measured using rat-specific radioimmunoassay (Millipore, St. Charles, MO). Adiponectin was measured using a mouse-specific radioimmunoassay (Millipore, St. Charles, MO). Urine metabolites were measured as described previously [13], [14]. Results were reported in µM, and converted to g/d based on total 24 h urine volume.

### Glucose Tolerance Tests

For GTT, the rats were fasted overnight (12–15 hours) at 14 and 27 weeks of age. Animals were injected with 2 mg/g body weight of 50% D-glucose for GTT through oral gavage. Tail vein blood glucose levels were measured at 0, 15, 30, 60, and 120 minutes after injection using OneTouch Ultra Meter.

### Quantitative PCR

RNA was extracted from liver tissue of 9 week old animals using TRIzol reagent (Invitrogen, CA). cDNA was generated using high-capacity cDNA Archive Kit (SuperScript™ III Reverse Transcriptase, Invitrogen). mRNA of indicated genes was assessed by reverse transcription PCR (iCycler, BioRad) and normalized to TATA-Box binding protein (TBP) as described previously [15]. For RT-PCR, Absolute blue qPCR premix (Fisher Scientific) was mixed with each primer (Table 1).

**Table 1:** Primers used for quantitative PCR.

Gene name	*Forward 5′→3′*	*Reverse 5′→3′*
***G6pc***	***AGCTCCGTGCCTCTGATAAA***	***AAAGTGAGCCGCAAGGTAGA***
***PcK1***	***CCCAGGAGTCACCATCACTT***	***TTCGTAGACAAGGGGGACAC***
***Srebp-1c***	***GTTAACGTGGGTCTCCTCCG***	***TGATGCCTGCGGTCTTCATT***
***Tbp***	***TATAATCCCAAGCGGTTTGC***	***CAGCCTTATGGGGAACTTCA***

### Quantitative histology

Kidneys were fixed in 10% buffered formalin for at least 24 hours. A longitudinal 4–5 mm section was made on the midline extending from hilus to cortex on the opposite side. Sectioned tissues were processed routinely and embedded in paraffin blocks. Four uM sections from each block were made and slides were stained with Hematoxylin & Eosin and Periodic acid-Schiff.

A scoring system was developed for evaluating renal disease on the histologic sections of kidneys. A 0 or 1 grade was given based on the presence or absence, respectively, of the following changes; glomerular sclerosis, tubular dilation, intratubular protein, attenuation of tubular epithelium, tubular epithelial proliferation, tubule basement membrane thickening, mineralization, interstitial fibrosis, inflammation, pelvic dilation, and bacteria. In addition, these parameters (glomerular sclerosis, tubular dilation, intratubular protein, attenuation of tubular epithelium, tubular epithelial proliferation, tubule basement membrane thickening, mineralization, and inflammation) were also graded 1 to 4 based on area of involvement, in increments of 25% respectively. A separate score for overall area of all kidney changes (0 to 4) was also done in increments of 25% respectively.

Preliminary scoring of 15 week old animals indicated relatively little pathology. Thus, we scored a much larger number of kidneys from 28 week old fatty Zucker and fatty congenic animals.

### Data Analysis

Phenotype data are reported as mean (SE) and analyzed using statistical software from SAS (JMP version 10.0, SAS Institute Inc.). Absolute P-values at p = 0.05 or less are reported and those meeting the criteria for significance using the False Discovery Rate [16] are indicated by bold type. Repeated measures analysis was done by either MANOVA when doing 3-way analysis or Residual Maximum Likelihood when doing 2-way analysis and correlation analysis used Spearman's Rank Correlation. Comparisons of quantitative PCR data between the two groups were made using the two-sample (independent groups) t-test. Specific information for each analysis is provided in the legends for each table or figure.

## Results

We performed two related experiments, a cross-sectional study where animals were sacrificed at 9, 15, 24 and 28 weeks and a longitudinal study where non-lethal phenotypes were collected for a repeated measures analysis with sacrifice data at 28 weeks.

### Cross-sectional analysis of protection from disease phenotypes by the congenic donor region

Four cohorts of male rats, lean and fatty congenic, as well as lean and fatty ZUC animals, were sacrificed at 9, 15, 24 and 28 weeks for measurement of internal organ weights and collection of tissues for further analysis (2). Overall body weight for fatty ZUC and congenic was similar at 15 weeks, but by 28 weeks fatty congenics weighed more than fatty ZUC rats (Table 2). Three fat depots (mesenteric, gonadal and retroperitoneal) were weighed. Fat depots in fatty ZUC rats did not differ at 9 weeks from fatty congenics; by 15 weeks the fat depots in fatty ZUC rats were heavier than in congenics; and by 28 weeks only mesenteric white adipose tissue was heavier in fatty ZUC than congenic. ZUC rats have heavier kidneys at 15 and 28 weeks and heavier livers at 9 and 15 weeks than fatty congenics. Fatty ZUC and congenics have less gastrocnemius muscle mass than lean rats (Table 3). When gastrocnemius muscle mass is expressed as percent of body weight, there is a Chr 1 genotype difference with lean ZUC rats having less muscle mass than lean congenics.

**Table 2:** Body and organ weights at sacrifice in Zucker lean *Lepr^+Ste^* and fatty *Lepr^faSte^* rats with either Brown Norway (BN) or Zucker (ZUC) Chr 1 congenic region at 9, 15 or 28 weeks of age. Data are means ± SE.

Age	9 weeks				15 weeks				28 weeks			
*Lepr* genotype	*Lepr^+Ste^*		*Lepr^faSte^*		*Lepr^+Ste^*		*Lepr^faSte^*		*Lepr^+Ste^*		*Lepr^faSte^*	
Chr 1 genotype	BN	ZUC	BN	ZUC	BN	ZUC	BN	ZUC	BN	ZUC	BN	ZUC
Number	11	11	12	12	10	11	11	13	12	18	13	16
Body weight (g)	216±5^I^	266±6^H^	340±6^FG^	359±6^FG^	317±8^G^	371±5^F^	511±12^CD^	527±9^BC^	416±6^E^	475±4^D^	663±13^A^	557±12^B^
Liver (g)	5.74±0.13^H^	7.55±0.20^GH^	11.2±0.2^EF^	14.2±0.4^CD^	7.44±0.21^GH^	8.85±0.52^FG^	15.2±0.5^C^	22.7±0.4^B^	9.30±0.21^FG^	11.8±0.2^DE^	23.6±0.7^AB^	25.3±1.2^A^
Rt GWAT (g)	0.54±0.04^F^	0.92±0.06^EF^	3.65±0.13^CD^	4.16±0.15^C^	1.09±0.04^EF^	1.62±0.10^E^	5.83±0.32^B^	7.72±0.23^A^	1.54±0.06^E^	3.09±0.09^D^	8.01±0.25^A^	8.23±0.34^A^
RWAT (g)	0.123±0.012^E^	0.274±0.036^E^	2.01±0.13^D^	2.12±0.08^D^	0.346±0.015^E^	0.534±0.032^E^	4.44±0.22^C^	5.58±0.27^B^	0.653±0.046^E^	1.54±0.06^D^	10.5±0.3^A^	10.1±0.4^A^
MWAT (g)	0.55±0.06^F^	1.33±0.08^EF^	5.57±0.26^D^	7.02±0.29^D^	1.51±0.07^EF^	2.57±0.13^E^	9.47±0.67^C^	12.33±0.43^AB^	2.88±0.13^E^	5.81±0.22^D^	12.08±0.34^B^	13.59±0.43^A^
Total Kidney (g)	1.54±0.03^F^	1.88±0.03^EF^	2.19±0.04^DE^	2.53±0.21^CD^	1.98±0.05^DEF^	2.21±0.03^DE^	2.80±0.07^C^	3.86±0.06^B^	2.53±0.07^CD^	2.89±0.05^C^	4.32±0.14^B^	6.50±0.99^A^

Three-way ANOVA effect						
	*Lepr* genotype	Chr 1 genotype	*Lepr*×Chr 1 interaction	Age	Age×*Lepr* interaction	Age×Chr 1 interaction
Body weight	**<0.0001**	**0.0028**	**<0.0001**	**<0.0001**	**<0.0001**	**<0.0001**
Liver	**<0.0001**	**<0.0001**	**0.0013**	**<0.0001**	**<0.0001**	**0.0097**
Rt GWAT	**<0.0001**	**<0.0001**	0.8209	**<0.0001**	**<0.0001**	0.0359
RWAT	**<0.0001**	**0.0035**	0.5827	**<0.0001**	**<0.0001**	**0.1525**
MWAT	**<0.0001**	**<0.0001**	0.3495	**<0.0001**	**<0.0001**	**0.0462**
Total Kidney	**<0.0001**	**<0.0001**	**<0.0001**	**<0.0001**	**<0.0001**	**<0.0001**

AN length = anal-nasal length. Rt GWAT = right gonadal adipose tissue. RWAT = retroperitoneal white adipose tissue. MWAT = mesenteric white adipose tissue. Data analysis is by three-way ANOVA (Expected Means Squares approach) with mean comparisons by Tukeys HSD: means not sharing superscript are different at p≤0.05. P values in bold are significant at p = 0.05 when corrected for multiple testing using the False Discovery Rate.

**Table 3:** Body and Gastrocnemius muscle weights at sacrifice in Zucker lean *Lepr^+Ste^* and fatty *Lepr^faSte^* rats with either Brown Norway (BN) or Zucker (ZUC) Chr 1 congenic region at 28 weeks of age.

Lepr genotype	*Lepr^+Ste^*		*Lepr^faSte^*		Two-way ANOVA		
Chr 1 genotype	BN	ZUC	BN	ZUC	*Lepr* genotype	Chr 1 genotype	Interaction
Number	8	8	14	9			
Body weight (g)	405±10 ^B^	437±6 ^B^	629±10 ^A^	637±16 ^A^	**<0.0001**	0.0908	0.3108
Gastrocnemius (g)	2.44±0.07 ^A^	2.41±0.04 ^A^	2.08±0.05 ^B^	1.96±0.04 ^B^	**<0.0001**	0.1469	0.3943
Gastrocnemius % of BW	0.604±0.004 ^A^	0.552±0.009 ^B^	0.331±0.005 ^C^	0.309±0.008 ^C^	**<0.0001**	**<0.0001**	0.0342

Data are means ± SE.

Data analysis is by two-way ANOVA (Expected Means Squares approach) with mean comparisons by Tukeys HSD: means not sharing a superscript are different at p≤0.05. P values in bold are significant at p = 0.05 when corrected for multiple testing using the FDR.

**Figure 1:**
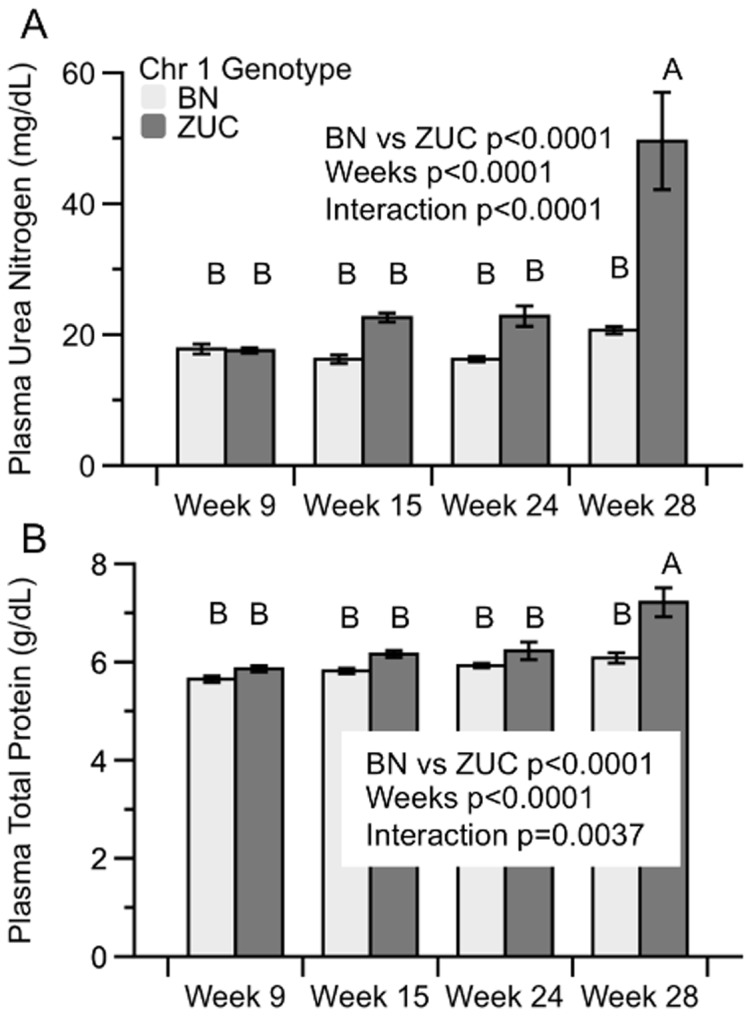
Plasma Urea Nitrogen (mg/dL) and Total Protein (g/dL) in Zucker Lepr^faSte^ rats with either Brown Norway (BN) or Zucker (ZUC) Chr 1 congenic region at 9, 15, 24 or 28 weeks of age. Data are mean ± SE, N = 10 each group. Analysis by 2-way ANOVA with mean comparisons by Tukey's HSD.

Fasting plasma was measured for indicators of renal disease, type 2 diabetes, and liver and muscle damage in fatty *Lepr^faSte^* male rats (Table 4 and Figure 1). Plasma urea nitrogen was 2-fold higher in ZUC strain than congenic strain rats by 28 weeks. There were no statistically significant differences of plasma total protein, glucose, urea nitrogen or creatinine between congenics and ZUC at weeks 9,15 and 24 but at week 28 all these variables were significantly higher in the fatty ZUC group. Total cholesterol and triglycerides were higher in fatty ZUC than in fatty congenics at all ages but large variances in these phenotype measurements reduced significance levels. Plasma albumin was significantly lower in 24-week ZUC strain than in congenics. Alanine aminotransferase, an indicator of liver damage, was only significantly higher in ZUC at 15 weeks.

**Table 4:** Plasma analysis of Zucker fatty *Lepr^faSte^* rats with either Brown Norway (BN) or Zucker (ZUC) Chr 1 congenic region at 9, 15, 24, or 28 weeks.

Age	9 weeks		15 weeks		24 weeks		28 weeks		Two-way ANOVA effect		
Chr 1 genotype	BN	ZUC	BN	ZUC	BN	ZUC	BN	ZUC	Chr 1 genotype	Age	Chr 1× Age
Number	10	10	10	10	10	10	10	10			
Albumin (gdL)	4.16±0.07^A^	4.10±0.04^AB^	3.74±0.06^BC^	3.43±0.01^CD^	3.25±0.05^D^	2.63±0.11^E^	2.79±0.11^E^	2.61±0.09^E^	**<0.0001**	**<0.0001**	0.0106
Alanine Aminotransferase (U/L)	51.6±2.4^BC^	60.9±3.3^B^	45.4±1.7^BCD^	81.3±8.0^A^	38.8±1.9^CD^	45.9±5.6^BCD^	31.8±2.1^D^	34.3±3.1^CD^	**<0.0001**	**<0.0001**	**0.0003**
Cholesterol (mg/dL)	94.6±3.0^D^	108±3^D^	116±5^CD^	209±19^BCD^	194±10^BCD^	357±75^AB^	328±25^ABC^	490±109^A^	**0.0023**	**<0.0001**	0.3650
Triglyceride (mg/dL)	170±12^C^	311±14^ABC^	307±31^BC^	846±180^ABC^	547±49^ABC^	1864±614^AB^	1290±219^ABC^	1869±733^A^	0.0119	**0.0012**	0.4152
Creatinine (mg/dL)	0.196±0.006^B^	0.176±0.005^B^	0.232±0.007^B^	0.236±0.007^B^	0.244±0.008^B^	0.252±0.020^B^	0.281±0.013^B^	0.656±0.115^A^	**0.0004**	**<0.0001**	**<0.0001**
Glucose (mg/dL)	183±9^BC^	142±7^C^	186±11^B^	171±5^BC^	169±4^BC^	171±11^BC^	181±3^BC^	241±19^A^	0.8197	**<0.0001**	**<0.0001**
Lipemia Index	0^C^	0^C^	0.2±0.2^C^	2.4±0.2^AB^	1.8±0.4^B^	2.1±0.5^AB^	2.9±0.1^A^	2.6±0.2^AB^	**0.0024**	**<0.0001**	**<0.0001**

Samples taken at sacrifice. Data are means ± SE.

Data analysis is by two-way ANOVA (Expected Means Squares approach) with mean comparisons by Tukeys HSD: means not sharing a superscript are different at p≤0.05. P values in bold are significant at p = 0.05 when corrected for multiple testing using the FDR.

### Longitudinal study of disease progression

The second experiment was a longitudinal repeated measures study in which 4 cohorts of male lean and fatty congenic and ZUC strains were subjected to survival phenotyping at 9, 15, 24 and 28 weeks. Urine volume, body weight, food intake, and urinary albumin excretion (UAE) were measured (Table 5). Urine volume was 2-fold higher in fatty ZUC compared with fatty congenic at 9 weeks, and then became almost 3-fold higher for weeks 15 and 28. Fatty congenics had no significant change in urine volume throughout the study. The interaction of time*congenic*ZUC for urine volume was highly significant. The congenic strain had a larger fold effect on urinary albumin excretion (UAE) in lean than fatty animals – up to more than 10-fold in lean and 1 to 4-fold in fatty animals. By 28 weeks fatty congenic and fatty ZUC strains have essentially identical UAE values.

**Table 5:** Body weight, food intake, urine volume and urinary albumin excretion in the longitudinal study of Zucker *Lepr^+Ste^* and *Lepr^faSte^* rats with either Brown Norway (BN) or Zucker (ZUC) Chr 1 congenic region at 9, 15, 24 and 28 weeks.

Age	9 weeks				15 weeks				24 weeks				28 weeks			
*Lepr* genotype	*Lepr^+Ste^*		*Lepr^faSte^*		*Lepr^+Ste^*		*Lepr^faSte^*		*Lepr^+Ste^*		*Lepr^faSte^*		*Lepr^+Ste^*		*Lepr^faSte^*	
Chr 1 genotype	BN	ZUC	BN	ZUC	BN	ZUC	BN	ZUC	BN	ZUC	BN	ZUC	BN	ZUC	BN	ZUC
Number	10	18	11	11	10	18	11	11	10	18	11	11				
Body weight (g)	243±5	257±3	343±15	355±8	348±5	388±3	555±8	558±6	418±6	473±4	650±12	600±9	431±6	489±4	667±13	569±14
Food intake (g/d/BW)	0.37±0.01	0.41±0.01	0.44±0.01	0.51±0.01	0.30±0.01	0.30±0.01	0.28±0.01	0.38±0.01	0.25±0.01	0.25±0.01	0.22±0.00	0.31±0.02	0.26±0.00	0.25±0.00	0.23±0.01	0.24±0.01
Urine volume (ml)	8.6±0.6	15.1±1.1	23.8±1.7	46.6±5.1	10.3±0.6	17.4±1.2	27.3±4.8	95.1±10.9	14.6±1.1	19.1±0.8	30.6±8.2	123±16	16.5±1.1	19.6±0.8	33.4±9.0	108±14
Urinary albumin excretion (mg/d)	0.57±0.07	0.79±0.10	3.14±0.36	5.32±0.83	0.82±0.08	2.17±0.40	50.7±6.5	146±23	1.34±0.15	12.6±4.3	324±58	564±93	1.94±0.37	30.0±7.0	490±72	523±127

Main and Repeated Measures Effects							
	*Lepr* genotype	Chr 1 genotype	*Lepr*×Chr 1 interaction	Age (weeks)	Age×*Lepr* interaction	Age×Chr 1 interaction	Age×*Lepr*×Chr 1 interaction
Body weight	**<0.0001**	0.1689	**<0.0001**	**<0.0001**	**<0.0001**	**<0.0001**	**<0.0001**
Food intake	**<0.0001**	**<0.0001**	**<0.0001**	**<0.0001**	**<0.0001**	**<0.0001**	**<0.0001**
Urine volume	**<0.0001**	**<0.0001**	**<0.0001**	**<0.0001**	0.3797	0.0244	**<0.0001**
Urinary albumin excretion	**<0.0001**	**<0.0001**	**0.0010**	**<0.0001**	**<0.0001**	**0.0006**	**<0.0001**

Data are means ± SE.

Group size: *Lepr^+Ste^* BN = 10 and ZUC = 18; *Lepr^faSte^* BN = 11 and ZUC = 11. Repeated measures data analyzed by the Expected Mean Square Approach (JMP). Data log transformed before analysis. P values in bold are significant at p = 0.05 when corrected for multiple testing using the False Discovery Rate.

Daily food intake per gram body weight was calculated at weeks 9, 15, 24, and 28 (Table 5). There is a more prominent *Lepr* genotype effect on food intake at week 9 compared to the later ages. There was a significant reduction of daily food intake in all four groups over the course of the experiment, which was more pronounced in ZUC than congenic strain animals.

### Urine metabolites

We next measured urinary metabolites (n = 37) of Zucker fatty *Lepr^faSte^* male rats using ^1^H NMR spectroscopy, and converted into mass/d based on total urinary volume (Table 6). Ten metabolites were determined to have significant genotype effects, 19 to have significant effects of time, and 9 were significant for both time and genotype. Table 6 shows those with significant genotype effects. All remaining metabolites are listed in the footnote. The nine metabolites significant for both time and genotype were glucose, urea, valine, leucine, alanine, creatine, cadaverine, myo-inositol and 2-oxoglutarate. Urea loss showed a highly significant genotype effect where ZUC strain lost more than the congenic strain. For example at 15 weeks ZUC strain rats lost twice as much urea per day in urine as congenic strain animals. Urinary glucose loss is complex, with an increase from 9 to 15 weeks followed by a decrease from 15 to 28 weeks, but with overall 4–10 fold higher fold (4–10) levels in ZUC compared with the congenic strain. Myo-inositol, derived from glucose, shows a modest increase with age in ZUC, and is 2 to 6-fold higher in ZUC than the congenic strain.

**Table 6:** Urine metabolites in the longitudinal study of Zucker fatty *Lepr^faSte^* rats with either Brown Norway (BN) or Zucker (ZUC) Chr 1 congenic region at 9, 15, 24 and 28 weeks.

Age	9 weeks		15 weeks		24 weeks		28 weeks		p-values	
									Strain differences	Age differences
Congenic genotype	BN	ZUC	BN	ZUC	BN	ZUC	BN	ZUC		
2-Oxoglutarate (mg/d)	41.4±3.6	49.1±4.1	42.6±3.6	75.4±4.8	44.2±2.9	75.1±4.2	46.3±4.1	48.9±5.3	**0.0004**	**0.0005**
Alanine (µg/d)	396±24	546±40	456±23	786±38	565±50	1127±115	603±61	1737±342	**0.0003**	**<0.0001**
Cadaverine (µg/d)	420±32	337±27	564±30	530±33	626±26	416±35	656±51	439±41	**0.0005**	**<0.0001**
Creatine (µg/d)	245±14	275±23	413±37	1025±250	1370±276	5042±470	1815±263	6566±1428	**<0.0001**	**<0.0001**
Fumarate (µg/d)	986±106	1205±94	979±90	1711±57	1010±72	1802±136	1200±110	1344±160	**0.0003**	0.0221
Glucose (mg/d)	791±207	2482±402	1155±433	6377±868	606±488	6005±855	566±543	3716±923	**<0.0001**	**0.0018**
Isoleucine (µg/d)	73.5±6.8	91.5±7.5	92.1±6.1	117.7±15.5	102.7±7.7	140.7±16.7	120.7±13.5	159.2±17.4	0.0160	**<0.0001**
Leucine (µg/d)	178±17	206±21	246±29	326±21	278±20	389±31	298±33	512±56	**0.0007**	**<0.0001**
Myo-Inositol (mg/d)	3.0±0.4	8.2±1.0	3.1±0.6	13.1±1.3	2.5±0.9	15.9±1.4	2.9±1.1	13.9±1.9	**<0.0001**	**0.0084**
Urea (mg/d)	451±46	657±59	548±72	1136±85	507±58	916±53	507±58	746±61	**<0.0001**	**<0.0001**
Valine (µg/d)	166±12	216±14	218±11	327±15	245±18	369±21	267±22	460±48	**<0.0001**	**<0.0001**

Data are means ± S.E. Repeated measures analysis by Residual Maximum Likelihood. P values in bold are significant at p = 0.05 when corrected for multiple testing using the False Discovery Rate.

* Hippurate calculated using molecular weight for Hippuric acid (179).

Metabolites with no significant time or genotype effects: methylamine, NN Dimethylglycine, succinate, trimethylamine, trimethylamine oxide, creatinine, dimethylamine, formate, lactate, choline, cis-aconitate, allantoin, citrate, hippurate, Trigonelline. Metabolites with a significant age dependent increase: pyruvate, creatinine, glycerophosphocholine, threonine, leucine, trans-aconitate, acetate, betaine. Metabolites with a significant age dependent decrease: taurine, hypoxanthine, 1-methylnicotinamide, 3-indoxylsulfate.

The urinary branched chain amino acids, valine and leucine, were higher in ZUC rats and increased in both strains with age. However, while isoleucine showed an age dependent increase, it only showed a modest genotype effect. Both creatine and cadaverine, a diamine, had significant strain and age effects. Creatine increased by 7-fold in the congenic strain and 20-fold in the ZUC strain, while cadaverine showed a much smaller increase. Cadaverine was the only metabolite with a statistically significant genotype effect where congenics lost more per day than the ZUC strain. Urinary creatine may be derived from diet or muscle. Since all rats are eating the same diet, then urine differences in this analysis are more likely due to loss from muscle.

Spearman's rank correlations between urinary metabolites and volume at 9 and 28 weeks were calculated in *Lepr^faSte^* rats because 9 weeks captures animals while relatively healthy and the last time point captures animals that are much sicker. Our goal was to determine if any metabolites predict volume or have correlations that suggest that metabolites are being lost from specific pathways. Correlations for week 9 of Zucker *Lepr^faSte^* male rats calculated pairwise are shown in Table 7A. Urine volume is correlated with many metabolites, such as citric acid cycle metabolites, amino acids and urea in the congenic strain, but is not correlated with any metabolites in ZUC rats. Correlations between metabolites in 28 week urine are shown in Table 7B with correlations in congenics above the diagonal and correlations in ZUC rats below the diagonal. At 28 weeks, only urea, myo-inositol and volume are associated with each other in both ZUC and congenic rats. At both 9 and 28 weeks there are many more correlations in the congenics than in ZUC, including some involving citric acid cycle metabolites such as citrate, fumarate and 2-oxoglutarate.

**Table 7:** Correlations of selected urine metabolites at weeks 9 and 28 in *Lepr^faSte^* rats with either Chr 1 Brown Norway (BN) or Zucker (ZUC) genotype.

	Vol	2-Oxo	Ala	Allan	Cit	Cre	Fum	Glu	Hipp	Iso	Leu	Myo	Trans	Tri	Urea	Val
Vol	-	0.8909 0.0005	0.9152 0.0002	0.9394 <0.0001	0.7697 0.0092	0.8788 0.0008	0.8667 0.0012	0.8061 0.0049		0.9394 <0.0001	1.0000 <0.0001	0.8667 0.0012	0.8061 0.0049	0.9394 <0.0001	0.9515 <0.0001	0.9515 <0.0001
2-Oxo		-	0.8061 0.0045	0.9879 <0.0001	0.7818 0.0075	0.7939 0.0061	0.9879 <0.0001		0.7818 0.0075	0.9030 0.0003	0.8909 0.0005	0.9879 <0.0001	0.9636 <0.0001	0.9515 <0.0001	0.8424 0.0022	0.9152 0.0002
Ala			-			0.9879 <0.0001	0.8182 0.0038	0.9030 0.0003		0.9273 0.0001	0.9152 0.0002	0.8182 0.0038	0.7697 0.0092	0.9030 0.0003	0.9758 <0.0001	0.9636 <0.0001
Allan				-		0.9758 <0.0001	0.8788 0.0008	0.9152 0.0002	0.7939 0.0061	0.9394 <0.0001	0.9394 <0.0001	0.8788 0.0008	0.8303 0.0029	0.9394 <0.0001	0.9879 <0.0001	0.9879 <0.0001
Cit		0.9394 <0.0001			-		0.7697 0.0092		0.8545 0.0016	0.8424 0.0022	0.7697 0.0092	0.7697 0.0092		0.8061 0.0049		0.8182 0.0038
Cre						-	0.8061 0.0049	0.9152 0.0002	0.7697 0.0092		0.8788 0.0008	0.8061 0.0049	0.7818 0.0075	0.8909 0.0005	0.9515 <0.0001	0.9515 <0.0001
Fum		0.8303 0.0029			0.8545 0.0016		-	0.7818 0.0075	0.7939 0.0061	0.8909 0.0005	0.8667 0.0012	1.0000 <0.0001	0.9515 <0.0001	0.9394 <0.0001	0.8545 0.0016	0.9273 0.0001
Glu								-	0.7939 0.0061	0.8061 0.0049	0.8061 0.0049	0.7818 0.0075		0.8061 0.0049	0.8909 0.0005	0.9030 0.0003
Hipp									-			0.7939 0.0061	0.7939 0.0061			0.7818 0.0075
Iso										-	0.9394 <0.0001	0.8909 0.0005	0.8667 0.0012	0.9758 <0.0001	0.9152 0.0002	0.9636 <0.0001
Leu											-	0.8667 0.0012	0.8061 0.0049	0.9394 <0.0001	0.9515 <0.0001	0.9515 <0.0001
Myo		0.8788 0.0008			0.8182 0.0038							-	0.9515 <0.0001	0.9394 <0.0001	0.8545 0.0016	0.9273 0.0001
Trans								0.8667 0.0012					-	0.9394 <0.0001	0.7818 0.0075	0.8667 0.0012
Tri		0.8061 0.0049			0.9030 0.0003									-	0.9152 0.0002	0.9636 <0.0001
Urea		0.7939 0.0061						0.9273 0.0001			0.7697 0.0092	0.8424 0.0022			-	0.9758 <0.0001
Val		0.8788 0.0008			0.8181 0.0038					0.8061 0.0049				0.8182 0.0038		-
Vol	-		0.8936 0.0005	0.8632 0.0013						0.7927 0.0062	0.9512 <0.0001	0.7964 0.0058		0.9119 0.0002	0.9483 <0.0001	0.9423 <0.0001
2-Oxo		-	0.9030 0.0003		NEG 0.8303 0.0029	0.7697 0.0092	0.9636 <0.0001									
Ala			-				0.8424 0.0022				0.8571 0.0015			0.8182 0.0038		0.9394 <0.0001
Allan				-					0.8545 0.0016		0.7903 0.0065	0.8303 0.0029		0.9394 <0.0001	0.9273 0.0001	0.6797 0.0092
Cit					-		0.8303 0.0029			0.7842 0.0072						
Cre						-										
Fum							-									
Glu								-								
Hipp								0.7818 0.0075	-					0.7939 0.0061		
Iso										-	0.8415 0.0023					0.7720 0.0089
Leu											-	0.7842 0.0072		0.8146 0.0041	0.9240 0.0001	0.9301 <0.0001
Myo	0.9515 <0.0001							0.8424 0.0022				-		0.8424 0.0022	0.8061 0.0049	0.7939 0.0061
Trans													-			
Tri								0.7697 0.0092						-	0.8667 0.0012	0.8667 0.0012
Urea	0.8424 0.0022							0.8545 0.0016				0.9515 <0.0001		0.8061 0.0049	-	0.8545 0.0016
Val																-

Data are Spearman's correlation (rho) and p-value. Because N = 10 each group, the p values should be considered with caution. Congenics with BN genotype at Chr 1 are above the diagonal: rats with ZUC genotype are below the diagonal.

Vol = Urine volume; 2-Oxo = 2-Oxoglutarate; Ala = Alanine; Allan = Allantoin; Cit = Citrate; Cre = Creatine; Fum = Fumarate; Glu = Glucose; Hipp = Hippurate; Iso = Isoleucine; Leu = Leucine; Myo = *myo*-inositol; Trans = trans-Aconitate; Tri = Trigonelline; Urea = Urea; Val = Valine.

### Urinary proteins

**Figure 2:**
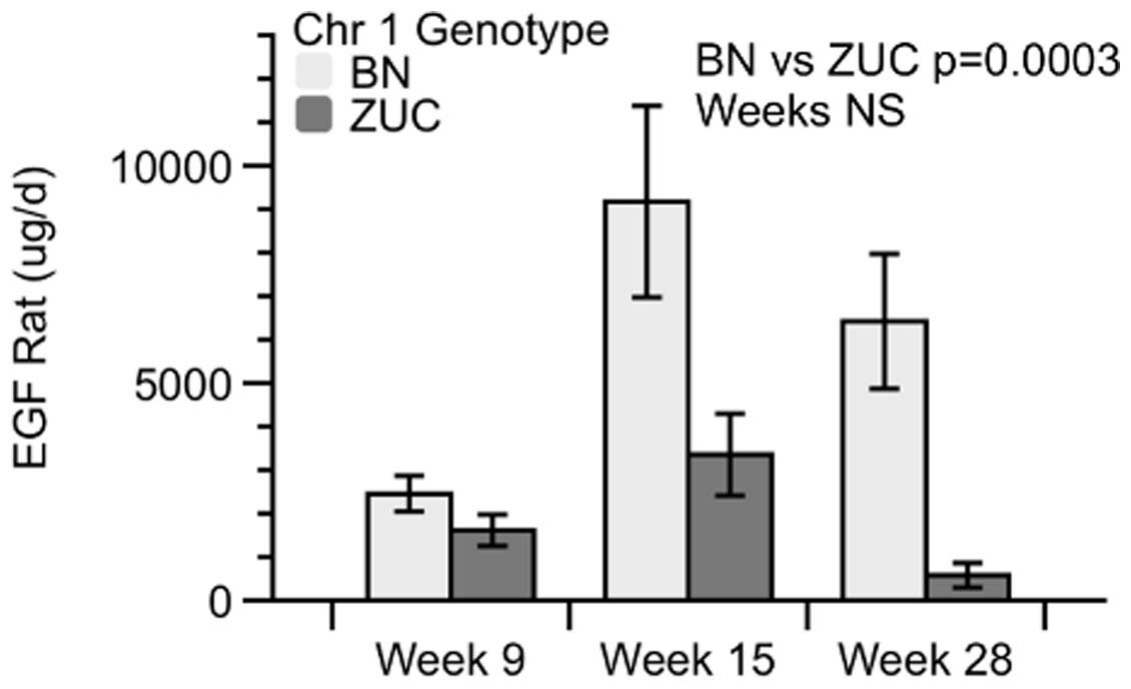
Urine Epidermal Growth Factor Rat (µg/d) in Zucker Lepr^faSte^ rats with either Brown Norway (BN) or Zucker (ZUC) Chr 1 congenic region at 9, 15, and 28 weeks of age. Data are mean ± SE, N = 6 each group. Repeated measures analysis by Residual Maximum Likelihood.

Tables 2–7 show in Zucker fatty *Lepr^faSte^* male rats, fatty congenics are significantly more protected from renal disease relative to the ZUC strain, yet both strains develop renal disease. The ZUC strain develops disease faster and more severely, but neither strain is as healthy as lean ZUC or lean congenic strains. Thus, urinary protein markers were measured to provide an indication of where kidneys were damaged. The KidneyMAP panel of 12 proteins was used to investigate damage to glomerulus, proximal and/or distal tubules. We compared fatty congenics to fatty ZUC at 9, 15, and 28 weeks in a repeated measures experiment (Table 8). The result from this analysis revealed seven proteins that showed significant age effects but not genotypic effects. Vascular endothelial growth factor A (VEGF) is reno-protective, whereas five of the age dependent proteins are controlled by the glomerulus or proximal tubules and one, calbindin, indicates distal tubule damage [17]. VEGF and beta-2-microglobulin both showed significant time effects in our data and by 28 weeks both proteins in ZUC rats were more than 3-fold higher than in congenic rats (Table 8). Both VEGF and beta-2-microglobulin showed a genotype effect in lean versus Zucker Diabetic Fatty (ZDF) rats in a previous study [18]. One protein, epidermal growth factor (EGF), showed significant genotype effects but no age effects (Figure 2). Total daily EGF was significantly decreased in fatty ZUC rats. EGF was found to have a genotype effect for lean versus ZDF with p<0.01 at 20 and 25 weeks in [18].

**Table 8:** Urinary protein markers from the KidneyMAP panel in Zucker fatty *Lepr^faSte^* rats with either Brown Norway (BN) or Zucker (ZUC) Chr 1 congenic region at 9, 15, and 28 weeks.

Age	9 weeks		15 weeks		28 weeks		p-value	
							Congenic genotype	Age
Congenic genotype	BN	ZUC	BN	ZUC	BN	ZUC		
Albumin Rat (mg/dl)	0.76±0.11	4.00±1.79	7.39±0.47	13.14±3.37	3.24±0.81	34.45±14.09	0.0402	0.0184
Beta-2-Microglobulin (µg/d)	263±33	368±71	212±18	1313±380	2204±219	7861±2067	0.0262	**<0.0001**
Calbindin (µg/d)	12.72±1.20	12.91±1.84	9.09±0.78	9.42±0.78	6.05±2.34	5.11±1.19	NS	**<0.0001**
Clusterin (µg/d)	5.47±0.64	4.73±1.01	10.63±0.76	48.65±21.03	69.37±16.99	341.78±83.26	0.0128	**0.0001**
Cystatin-C (µg/d)	4.91±0.23	6.67±0.79	5.74±0.24	10.60±1.83	10.06±1.44	29.23±7.57	0.0359	**0.0003**
Glutathione S-Transferase Alpha (ng/d)	96.7±8.2	150±35	71.8±1.6	131.6±29.2	163.8±25.8	403.8±109.1	0.0390	**0.0026**
Glutathione S-Transferase Mu (ng/d)	78.5±6.6	187.5±85.4	58.3±1.3	152.7±92.2	63.9±7.9	960.3±244.1	0.0079	0.0153
Kidney Injury Molecule-1 Rat (ng/d)	3.34±0.20	6.36±0.76	4.03±0.24	15.69±4.19	44.95±19.39	99.8±24.8	NS	**<0.0001**
Neutrophil Gelatinase-Associated Lipocalin (µg/d)	2.96±0.08	4.10±0.45	2.84±0.39	4.43±0.75	2.95±0.50	28.86±23.43	NS	NS
Osteopontin (ng/d)	119.2±2.9	171.7±22.9	45.8±3.5	76.8±11.9	49.7±6.3	566.3±206.1	0.0219	NS
Tissue Inhibitor of Metalloproteinases-1 Rat (ng/d)	65.0±6.4	78.9±13.8	161.0±14.5	288.1±38.4	354.9±153.6	662.9±54.9	0.0284	**<0.0001**
Vascular Endothelial Growth Factor A (pg/ml)	15.1±1.1	16.6±2.3	19.2±1.8	47.6±10.6	29.0±2.7	95.1±21.5	0.0104	**0.0004**

Data are means ± SE, N = 6 each group. Repeated measures analyses were done by Residual Maximum Likelihood (JMP). P–values in bold are significant when corrected for multiple testing using the False Discovery Rate.

### Quantitative histology

**Figure 3:**
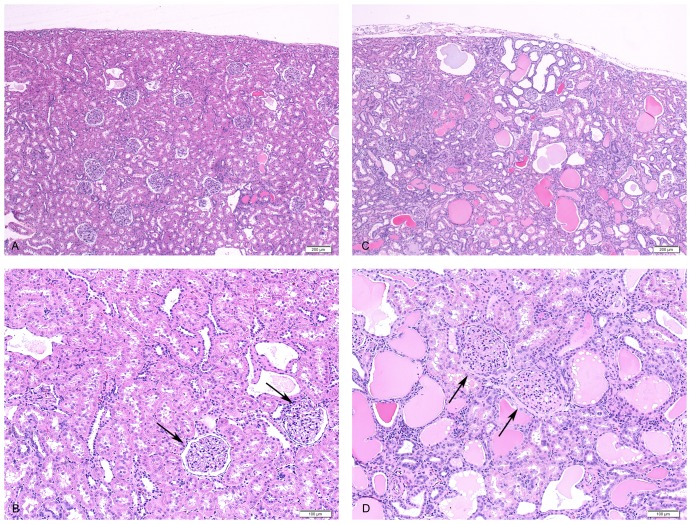
Histology of fatty congenic and ZUC animals at 28 weeks of age. High and low magnification of Hematoxylin and Eosin stained histologic sections from fatty congenic (A,B) and ZUC (C,D) kidneys. ZUC has an increase in dilated tubules and intraluminal protein (pink) compared to the congenic. In addition, ZUC (D) has sclerotic glomeruli (arrows) with thickened Bowman's capsule as compared to glomeruli (arrows) in the congenic (B).

Genotype effects on renal pathology were quantitated to determine if both glomeruli and tubules were equally affected. Light microscopic evaluation shows differences for both glomeruli and tubules in 28 week old fatty ZUC compared to fatty congenic (Figure 3). The glomerular basement membrane is thicker and intratubular volume is increased in the ZUC compared with fatty congenics. Table 9 provides results for quantitative histology for fatty ZUC and congenic at 28 weeks. These results reveal that the most significant genotype effects are for intratubular volume and protein, but area involved in glomerular sclerosis also has a highly significant genotype effect.

**Table 9:** Pathology scores in *Lepr^faSte^* male rats with either ZUC or Brown Norway chromosome 1 congenic region at 28 weeks.

Scored Pathology	ZUC (n = 14)	Congenic (n = 17)	Wilcoxin Z score
Sclerotic glomeruli area involved in increments of 25%	2.71±1.33	1.06±0.75	**0.0007**
Maximum glomerular score = 5	3.71±1.33	1.88±1.05	**0.0007**
Tubule dilation area involved in increments of 25%	2.86±0.86	1.41±0.51	**<0.0001**
Intratubular protein area involved in increments of 25%	2.29±0.61	1.29±0.47	**0.0002**
Maximum tubular score = 25	14.5±4.13	9.59±2.67	**0.0011**
Interstitial fibrosis	0.71±0.47	0.18±0.39	**0.0032**
Maximum cumulative score = 43	21.43±6.87	13.59±4.43	**0.0021**
Overall area of kidney with pathologic changes in increments of 10%	3±1.36	1.53±0.62	**0.0047**
Maximum overall area affected score = 4	3.28±0.91	1.53±0.62	**<0.0001**

Pathology scores expressed as means ± SD. Analysis by ANOVA and Wilcoxin rank scores. Z scores significant using False Discovery Rate are in bold. Only phenotypes with significant genotype effects are shown. All scored phenotypes are listed in methods.

### Type 2 diabetes

**Figure 4:**
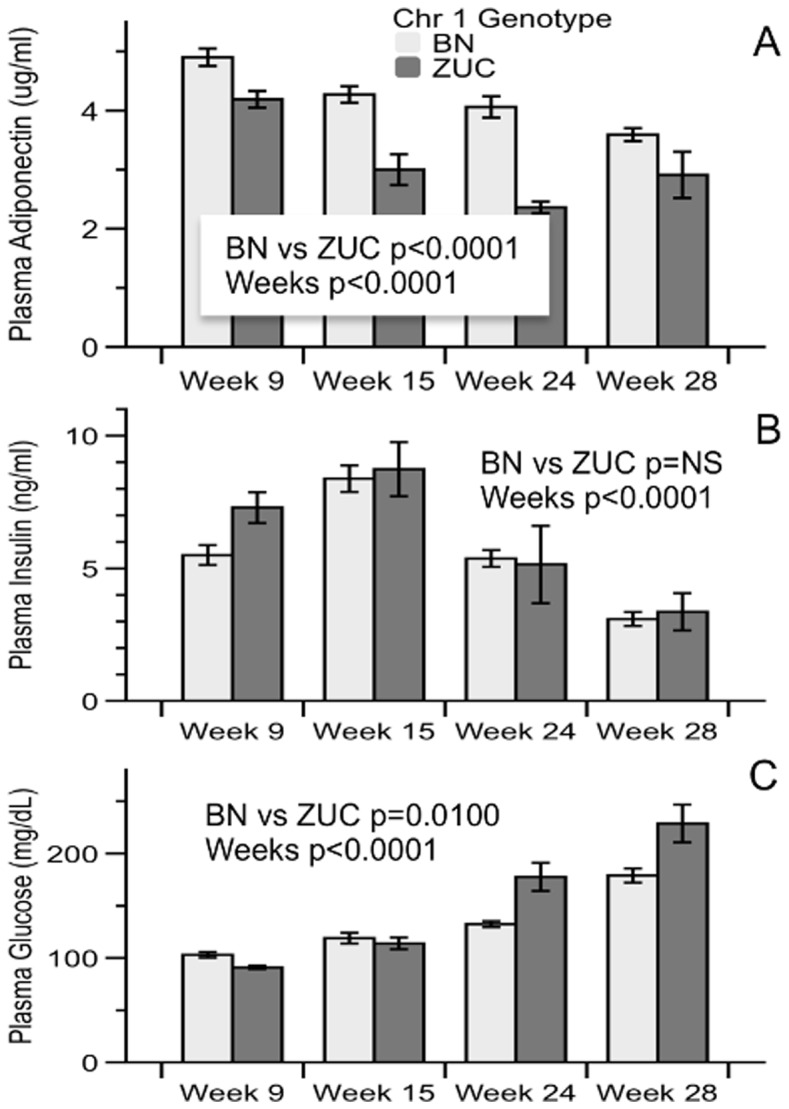
Fasting plasma Adiponectin (ug/mL), Insulin (ng/mL) and Glucose (mg/dL) in Zucker Lepr^faSte^ rats with either Brown Norway (BN) or Zucker (ZUC) Chr 1 congenic region at 9, 15, 24 or 28 weeks of age. Data are mean ± SE, BN N = 12: ZUC N = 7 for Adiponectin and Insulin, N = 12 for Glucose. Repeated measures analysis by Residual Maximum Likelihood.

**Figure 5:**
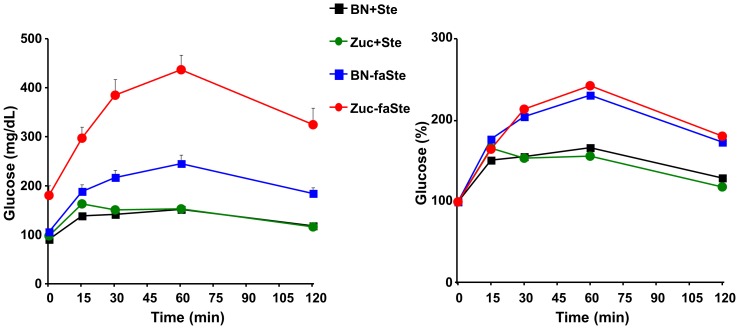
Glucose Tolerance Tests. Glucose Tolerance Tests were performed on 28-week old male lean and fatty ZUC (n = 18, 12 respectively) and lean and fatty congenic (n = 12, 13, respectively) as described in methods. Analyzed by Manova with measures repeated over time.

We showed (Table 6) that fatty ZUC lose much more glucose in urine than fatty congenics. We therefore tested if the congenic donor region influences the degree or progression of type 2 diabetes in fatty animals. We measured glucose, insulin and adiponectin levels in fasted plasma from animals at all ages. There was no genotype effect on insulin levels and a weak genotype effect on plasma glucose, but a highly significant increase of adiponectin in fatty congenics compared to fatty ZUC (Figure 4). Glucose tolerance tests (GTT) show that fasting glucose is higher in fatty ZUC than congenic (Figure 5). P-values for effect of *Lepr^faSte^* genotype, effect of congenic genotype, interaction between *Lepr^faSte^* genotype and congenic genotype and interaction between *Lepr^faSte^* genotype and time were all p<0.0001. There was no significant effect of time*congenic genotype interaction. When results are expressed as percentage change of initial fasting glucose levels there are no significant differences between ZUC and congenic, although fatty and lean animals are significantly different (Figure 5). We also evaluated the possibility that rates of gluconeogenesis exhibit genotype effects. Quantitative PCR was performed on liver samples from 9-week old fatty ZUC and congenic (Table 10). mRNA of phosphoenolpyruvate carboxykinase (PEPCK), and glucose-6-phosphatase (G-6-Pase) were significantly increased in fatty congenic compared to fatty ZUC.

**Table 10:** Quantitative PCR of liver mRNA.

*Gene*	Fold Congenic/ZUC ± SE	p-value
*G6pc*	2.4±0.26	0.008
*PcK1*	1.8±0.27	0.016

mRNA was extracted from 6 each livers of 9 week old animals.

## Conclusions

We used a homozygous congenic ZUC.BN-Chr1 (*D1Rat42-D1Rat90*) to determine effects of the chromosome 1 donor region on renal disease separate from the chromosome 4 *Lepr^faSte^* fatty mutation. The chromosome 1 congenic region reduces some renal disease phenotypes more than others. Quantitative histology indicates significant genotype effects on glomerular and tubular damage in 28 week old animals, with the most significant effects on intratubular volume. Although significant genotype effects were observed for UAE, both fatty strains show significant increases with age.

We used the Kidney MAP panel, which includes 7 proteins present in rat urine [17]. Previous work by other investigators used this panel to compare urine proteins in the Zucker Diabetic Fatty (ZDF) strain with lean controls [18]. Genotype effects but not time effects are presented in Table 4 of the paper by Togashi and Miyamoto [18]. The ZDF strain is related to the ZUC strain because both carry the *Lepr^fa^* mutation, but the strains have been separated for many years. Thus, other genes may have different alleles.

Many of the markers of glomerular and proximal tubule disease show age dependent increases in both ZUC.BN-Chr1 congenic and ZUC strain. However, one marker of distal tubule injury reached statistical significance for genotype effect. Levels of EGF decreased over time in the ZUC but not congenic strain, leading to a statistically significant genotype effect. This result is consistent with observations that lower EGF levels are correlated with kidney injury, perhaps because EGF contributes to repair from renal injury [18], [19]. Thus, both strains develop glomerular and tubular renal disease, but the ZUC.BN-Chr1 strain donor region has some protective effects which may be greatest in distal tubules.

Analysis of plasma total protein and albumin revealed significantly more total protein in ZUC at 28 weeks and less plasma albumin at 24 weeks. Although not directly measured, the difference between total protein and plasma albumin suggests increased globulins at 28 weeks in ZUC strain animals (Table 4 and Figure 1). Overall, these results are consistent with the hypothesis that both strains develop renal disease as indicated by increased UAE and glucose, but congenics have significantly less symptoms of disease than ZUC.

Phenotyping for type 2 diabetes also revealed variable effects of the congenic donor region. Significant effects were observed for urine glucose, fasting glucose and adiponectin. No significant effects were observed for GTT or fasting insulin. In addition, we observed increased expression of PEPCK and G6P in livers of congenics at 9 weeks. Causal genes for the genotype effects on type 2 diabetes could thus be expressed in liver, adipose or other tissues.

Our results are also consistent with the hypothesis that calories are being partitioned more towards fat accumulation in ZUC than in congenics. Evidence for this comes from many overlapping pieces of data. Body weights of fatty ZUC are lower than congenics at 28 weeks, but fat mass is the same or greater in ZUC. Although ZUC eat slightly more than congenics, their urinary urea loss is much higher than congenics, consistent with loss of lean mass from ZUC. And gastrocnemius muscle mass as a percent of body weight is higher in lean congenics than lean ZUC rats. These results suggest that analysis of total lean mass would reveal Chr 1 genotype effects.

Urinary cadaverine loss per day was higher in male fatty congenic than ZUC. Urine cadaverine has been correlated with gut microbiota, although this is mostly unexplored [20].

Previous results from our laboratory showed that food intake restriction reduces both renal disease and serum TG levels [4], [5]. Our current data confirm higher food intake in fatty ZUC than fatty congenic. Plasma TG levels are roughly correlated with UAE in that both plasma TG and UAE are higher in 9 and 24 week old ZUC than congenic, whereas UAE and TG are high but do not differ between strains at 28 weeks.

There are many positional candidates genes for kidney disease on rat chromosome 1. Rat chromosome 1 has several known renal disease genes (VPS10 domain receptor protein (Sorcs1 [9]), and Ras-related protein (Rab38 [8])), at least one gene that has been identified as a renal function gene in human genome wide association studies (Pancreatic secretory granule membrane major glycoprotein (Umod, [21]), orthologs of genes known to cause renal disease (Polycystic kidney disease 2-like 1 protein (Pkd2l1 [22])), and other genes that could plausibly influence renal disease (Aminopeptidase N precursor (Anpep [23]), Fumarylacetoacetase (Fah [24]), Aquaporin-11 (Aqp11 [25]), and Amiloride-sensitive sodium channel subunit gamma (Scnn1g [26]). Since we have previously demonstrated two QTL peaks for the albumin to creatinine ratio (ACR) in the BN donor region, one of which is quite broad, then there are at least two and probably more ACR genes in our congenic. In addition, there are many unannotated genes expressed in kidney that could influence congenic renal phenotypes. Thus, causal genes for our congenic model may include some of those listed above and others yet to be revealed as important.

We created a novel model of renal disease and type 2 diabetes in a fatty rat. Because we are using a congenic rat homozygous for the *Lepr^faSte^* mutation on chromosome 4, in fatty animals we have relatively small effects on fat pad weights from alleles on chromosome 1 (Table 2). Differences between congenic and ZUC are more pronounced when total fat pad mass is expressed as a percent of body weight because congenics have lower body weight than ZUC. At 28 weeks measured fat pad mass is 4.6% of body weight for fatty congenic and 5.7% for fatty ZUC. At 9 and 15 weeks these same comparisons show fatty ZUC is 3.9 and 3.8 -fold greater percent fat than lean, respectively. At these same ages percent of weight that is fat pads is 1.2 and 1.3-fold higher in fatty ZUC, respectively, than fatty congenic. Thus, we cannot rule out the possibility that type 2 diabetes and renal effects of the congenic donor region of fatty rats are secondary to a reduction of total or percent fat mass. However, claims that the diabetes in ZUC is secondary to fat mass effects would require that small differences in fat mass of ZUC compared to congenic have large effects on type 2 diabetes.

Our results are consistent with the hypothesis that some subphenotypes of the renal disease and type 2 diabetes related phenotypes of ZUC rats are caused by susceptibility alleles in ZUC rats and are not secondary to obesity. However, it is also true that renal disease is much worse in both fatty congenic and fatty ZUC than lean animals. Thus, obesity is the main cause of renal disease, but our results suggest that some phenotypes that appear to be due to *Lepr^faSte^* in ZUC are modified by genes on chromosome 1.

## References

[1] Kim K, Warden CH, Griffey SM, Vilches-Moure JG, Hansen S, et al (2010) Genes unlinked to the leptin receptor influence urinary albumin excretion in obese Zucker rats. Physiol Genomics 10.1152/physiolgenomics.90367.2008 PMC2869106 20159938

[2] Gades MD, van Goor H, Kaysen GA, Johnson PR, Horwitz BA, et al (1999) Brief periods of hyperphagia cause renal injury in the obese Zucker rat. Kidney international 56: 1779–1787. 10571786 10.1046/j.1523-1755.1999.00731.x

[3] Kasiske BL, Cleary MP, O'Donnell MP, Keane WF (1986) Effects of carbohydrate restriction on renal injury in the obese Zucker rat. American Journal Of Clinical Nutrition 44: 56–65. 3728350 10.1093/ajcn/44.1.56

[4] Gades MD, Van Goor H, Kaysen GA, Johnson PR, Horwitz BA, et al (1999) Brief periods of hyperphagia cause renal injury in the obese Zucker rat. Kidney Int 56: 1779–1787. 10571786 10.1046/j.1523-1755.1999.00731.x

[5] Stevenson FT, Wheeldon CM, Gades MD, van Goor H, Stern JS (2001) Hyperphagia as a mediator of renal disease initiation in obese Zucker rats. Obes Res 9: 492–499. 11500530 10.1038/oby.2001.64

[6] Wickramasinghe S, Rincon G, Islas-Trejo A, Medrano JF (2012) Transcriptional profiling of bovine milk using RNA sequencing. BMC Genomics 13: 45. 22276848 10.1186/1471-2164-13-45 PMC3285075

[7] Williams JM, Johnson AC, Stelloh C, Dreisbach AW, Franceschini N, et al (2012) Genetic variants in Arhgef11 are associated with kidney injury in the Dahl salt-sensitive rat. Hypertension 60: 1157–1168. 22987919 10.1161/HYPERTENSIONAHA.112.199240 PMC3505884

[8] Rangel-Filho A, Lazar J, Moreno C, Geurts A, Jacob HJ (2013) Rab38 modulates proteinuria in model of hypertension-associated renal disease. J Am Soc Nephrol 24: 283–292. 23291471 10.1681/ASN.2012090927 PMC3559491

[9] Lazar J, O'Meara CC, Sarkis AB, Prisco SZ, Xu H, et al (2013) SORCS1 contributes to the development of renal disease in rats and humans. Physiol Genomics 45: 720–728. 23780848 10.1152/physiolgenomics.00089.2013 PMC3742914

[10] Warden CH, Gularte-Merida R, Fisler JS, Hansen S, Shibata N, et al (2012) Leptin receptor interacts with rat chromosome 1 to regulate renal disease traits. Physiol Genomics 44: 1052–1062. 22968639 10.1152/physiolgenomics.00134.2011 PMC3615576

[11] Kessler MA, Meinitzer A, Wolfbeis OS (1997) Albumin blue 580 fluorescence assay for albumin. Anal Biochem 248: 180–182. 9177738 10.1006/abio.1997.2113

[12] Kessler MA, Meinitzer A, Petek W, Wolfbeis OS (1997) Microalbuminuria and borderline-increased albumin excretion determined with a centrifugal analyzer and the Albumin Blue 580 fluorescence assay. Clin Chem 43: 996–1002. 9191552

[13] Murdoch TB, Fu H, MacFarlane S, Sydora BC, Fedorak RN, et al (2008) Urinary metabolic profiles of inflammatory bowel disease in interleukin–10 gene-deficient mice. Anal Chem 80: 5524–5531. 18558774 10.1021/ac8005236

[14] Slupsky CM, Cheypesh A, Chao DV, Fu H, Rankin KN, et al (2009) Streptococcus pneumoniae and Staphylococcus aureus pneumonia induce distinct metabolic responses. J Proteome Res 8: 3029–3036. 19368345 10.1021/pr900103y

[15] Nagata N, Matsuo K, Bettaieb A, Bakke J, Matsuo I, et al (2012) Hepatic Src homology phosphatase 2 regulates energy balance in mice. Endocrinology 153: 3158–3169. 22619361 10.1210/en.2012-1406 PMC3380313

[16] Benjamini Y, Hochberg Y (1995) Controlling the False Discovery Rate: A Practical and Powerful Approach to Multiple Testing. Journal of the Royal Statistical Society Series B (Methodological) 57: 289–300.

[17] Dieterle F, Sistare F, Goodsaid F, Papaluca M, Ozer JS, et al (2010) Renal biomarker qualification submission: a dialog between the FDA-EMEA and Predictive Safety Testing Consortium. Nat Biotechnol 28: 455–462. 20458315 10.1038/nbt.1625

[18] Zeng F, Zhang MZ, Singh AB, Zent R, Harris RC (2007) ErbB4 isoforms selectively regulate growth factor induced Madin-Darby canine kidney cell tubulogenesis. Mol Biol Cell 18: 4446–4456. 17761534 10.1091/mbc.E07-03-0223 PMC2043549

[19] Zeng F, Singh AB, Harris RC (2009) The role of the EGF family of ligands and receptors in renal development, physiology and pathophysiology. Exp Cell Res 315: 602–610. 18761338 10.1016/j.yexcr.2008.08.005 PMC2654782

[20] Satink HP, Hessels J, Kingma AW, van den Berg GA, Muskiet FA, et al (1989) Microbial influences on urinary polyamine excretion. Clin Chim Acta 179: 305–314. 2714003 10.1016/0009-8981(89)90093-4

[21] Reznichenko A, Boger CA, Snieder H, van den Born J, de Borst MH, et al (2012) UMOD as a susceptibility gene for end-stage renal disease. BMC Med Genet 13: 78. 22947327 10.1186/1471-2350-13-78 PMC3495046

[22] Audrezet MP, Cornec-Le Gall E, Chen JM, Redon S, Quere I, et al (2012) Autosomal dominant polycystic kidney disease: comprehensive mutation analysis of PKD1 and PKD2 in 700 unrelated patients. Hum Mutat 33: 1239–1250. 22508176 10.1002/humu.22103

[23] Kotlo K, Hughes DE, Herrera VL, Ruiz-Opazo N, Costa RH, et al (2007) Functional polymorphism of the Anpep gene increases promoter activity in the Dahl salt-resistant rat. Hypertension 49: 467–472. 17242304 10.1161/01.HYP.0000256303.40359.38

[24] Luijerink MC, van Beurden EA, Malingre HE, Jacobs SM, Grompe M, et al (2004) Renal proximal tubular cells acquire resistance to cell death stimuli in mice with hereditary tyrosinemia type 1. Kidney Int 66: 990–1000. 15327392 10.1111/j.1523-1755.2004.00788.x

[25] Holmes RP (2012) The role of renal water channels in health and disease. Mol Aspects Med 33: 547–552. 22252122 10.1016/j.mam.2012.01.001 PMC6900978

[26] Haloui M, Tremblay J, Seda O, Koltsova SV, Maksimov GV, et al (2013) Increased renal epithelial na channel expression and activity correlate with elevation of blood pressure in spontaneously hypertensive rats. Hypertension 62: 731–737. 23959560 10.1161/HYPERTENSIONAHA.113.01295

